# Supplementary material for: Optimizing health service location in a highly urbanized city: Multi criteria decision making and P-Median problem models for public hospitals in Jeddah City, KSA
Source: PLoS One. 2024 Jan 2;19(1):e0294819. doi: 10.1371/journal.pone.0294819 (PMC10760729; doi:10.1371/journal.pone.0294819)
Supplement: Attachment — Submitted filename: Response Letter_PONE-D-23-14949R2.docx [file pone.0294819.s003.docx]

**Response to the editor comments**

Dear Academic Editor

I am pleased to resubmit to you my new paper titled: Optimizing health service location in a highly urbanized city: multi criteria -P-Median model for public hospitals in Jeddah city, KSA

We have carefully reviewed the editor’s comments and addressed almost them in the revised manuscript. All revised portions are marked in YELLOW in the revised manuscript, which we would like to submit for your kind consideration. The authors pay special thanks to the editor and reviewer because their comments and suggestions have greatly improved quality of the manuscript.

I hope that this paper can be accepted for your journal.

Best Regards

Prof. Abdulkader Murad

**Additional Editor Comments:**

**Comment 1:** Given the combination of the results and discussion sections, it is essential to provide comprehensive and critical discussions of the results obtained in this study. Such discussions are necessary to validate and justify the key findings of this research.

**Response 1:** Thank you for your comments. The comprehensive discussion of the study’s results validates the critical importance of addressing healthcare access disparities in urban areas, as revealed by the spatial distribution of resources in Jeddah. The selection of the fuzzy ‘And’ model, P-Median model, and the integration of the MC-P-Median model with fuzzy overlay is justified by their ability to optimize resource allocation, tackle uncertainty, and eliminate redundancy. The findings emphasize that while the current allocation is deemed feasible, further optimization is imperative to meet the healthcare demands of Jeddah’s growing population. These results have broader implications for urban healthcare planning globally, underscoring the need for data-driven, context-specific strategies and advanced spatial decision support systems to ensure equitable and efficient healthcare services as urban populations continue to expand. Please see pages 20-21 and lines 354-381.

**…. Added discussion….**

“One of the key takeaways from this study is the utilization of a GIS for healthcare planning. Integration of GIS technology with hospital locations enabled a detailed analysis of the distribution of healthcare resources and service demands. Combining P-Median model and fuzzy overlay techniques in a spatial support system addressed complex issues related to healthcare access and resource allocation. Results emphasized the importance of population density, the proximity of healthcare facilities, and travel times in healthcare planning. This approach recognized that healthcare accessibility is not solely dependent on number of hospitals but also on their strategic placement relative to population centers. Furthermore, the study also underscored significance of understanding local context, including factors like available resources, population density, and nature of demand locations when determining travel time thresholds. This adaptability and context-based decision-making are vital for effective healthcare planning.

The study asserts that integration of fuzzy overlay with MC-P-Median approach is superior to traditional methods, such as buffer analysis or distance-based catchment modelling. The findings related to the need for additional hospitals to cover all residents in Jeddah are a crucial finding and can help policy makers to enhance healthcare facilities situation. While the current allocation is deemed feasible, it is acknowledged that further optimization is necessary to ensure comprehensive healthcare coverage for the growing population. This raises questions about the scalability of the proposed models to other urban areas with increasing populations, emphasizing the need for ongoing research and adaptability in healthcare planning strategies.

This work has some limitations including its focus was solely on Jeddah city. This limitation may affect comprehensive understanding of healthcare needs and dynamics in the broader region. The discussion should have the implications for not accounting for potential demand from neighbouring areas and the importance of regional dynamics in healthcare planning. In conclusion, this study offers a comprehensive and innovative approach to allocating healthcare facility and optimizing in urban areas. However, the findings underscored the importance of data-driven and context-specific strategies in addressing complex issue associated with healthcare accessibility for growing urban populations.”

**Comment 2:** Regarding Figure 3-5, it is recommended to ensure consistency in the layout, such as positioning the mapping components in a similar fashion. Additionally, consider the possibility of combining these figures into one layout for clarity. Overlaying the locations of hospitals on these maps would enhance the reference points for readers, particularly when making geographical comparisons with Figure 1.

**Response 2:** We greatly appreciate the editor’s valuable input regarding the layout and presentation of our figures. We have agreed that consistency in the positioning of mapping components is essential for a coherent presentation. We have ensured that the layout of Figure 3-5 has been standardized to make it easier for readers to navigate and compare the information. Additionally, we have explored the possibility of combining these figures into one layout to enhance clarity and facilitate geographical comparisons, as suggested. We have also overlaid hospital locations on these maps to provide readers with clear reference points, which has improved the accessibility of our findings.

**Figure 3. a.** Number of beds in hospital derived by Kernel density. **b.** Population density in Jeddah as a measure of health demand. **c.** A provider-to-population result-based fuzzy overlay analysis. The public hospitals better serve locations in red, while locations in green need additional hospitals (The figure made with ArcGIS v.10.5 software (https://www.esri.com/en-us/arcgis/products/arcgis-desktop/resources)). [Shape file or base map that is freely available at https://www.diva-gis.org/gdata and do not have copyright restrictions.]

**Comment 3:** For the classification range, it is advisable to reconsider the current classes, which appear somewhat arbitrary (e.g., 6781.93 to 9040.88). A more reader-friendly and intuitive approach would be to use rounded ranges, such as 6500 to 9000, which will be easier for readers to comprehend.

**Response 3:** We appreciate the editor’s feedback regarding the classification range in our study. We agree that using more reader-friendly and rounded ranges, such as 6500 to 9000, could enhance the clarity and comprehension of our data. We have revisited the classification ranges and made the necessary adjustments to ensure that our readers can easily interpret the information in the revised manuscript.

**Figure 3. a.** Number of beds in hospital derived by Kernel density. **b.** Population density in Jeddah as a measure of health demand. **c.** A provider-to-population result-based fuzzy overlay analysis. The public hospitals better serve locations in red, while locations in green need additional hospitals (The figure made with ArcGIS v.10.5 software (https://www.esri.com/en-us/arcgis/products/arcgis-desktop/resources)). [Shape file or base map that is freely available at https://www.diva-gis.org/gdata and do not have copyright restrictions.]
